# Impact of early heparin therapy on mortality in critically ill patients with sepsis associated acute kidney injury: a retrospective study from the MIMIC-IV database

**DOI:** 10.3389/fphar.2023.1261305

**Published:** 2024-01-11

**Authors:** Zhi-Peng Zhou, Li Zhong, Yan Liu, Zhen-Jia Yang, Jia-Jia Huang, Da-Zheng Li, Yu-Hua Chen, Ying-Yi Luan, Yong-Ming Yao, Ming Wu

**Affiliations:** ^1^ Department of Infection and Critical Care Medicine, Health Science Center, Shenzhen Second People’s Hospital and First Affiliated Hospital of Shenzhen University, Shenzhen, China; ^2^ Department of Traditional Chinese Medicine, The First Affiliated Hospital, Guizhou University of Chinese Medicine, Guiyang, China; ^3^ Department of Nosocomial Infection Prevention and Control, Shenzhen Second People’s Hospital, Shenzhen, China; ^4^ Postgraduate Education, Shantou University Medical College, Shantou, China; ^5^ Department of Emergency Medicine, Shenzhen Second People’s Hospital, Shenzhen, China; ^6^ Department of Central Laboratory, Beijing Obstetrics and Gynecology Hospital, Capital Medical University, Beijing, China; ^7^ Trauma Research Center, Medical Innovation Research Department and Fourth Medical Center of the Chinese PLA General Hospital, Beijing, China

**Keywords:** heparin, sepsis-associated acute kidney injury, outcome, mortality, marginal structural Cox model

## Abstract

**Background:** Inflammatory-coagulation dysfunction plays an increasingly important role in sepsis associated acute kidney injury (SAKI). This study aimed to investigate whether early heparin therapy improves survival in patients with SAKI.

**Methods:** Patients with SAKI were identified from the Medical Information Mart for Intensive Care-IV database. The patients were divided into two groups: those who received heparin subcutaneously within 48 h after intensive care unit (ICU) admission and the control group, who received no heparin. The primary endpoint was ICU mortality, the secondary outcomes were 7-day, 14-day, 28-day, and hospital mortality. Propensity score matching (PSM), marginal structural Cox model (MSCM), and E-value analyses were performed.

**Results:** The study included 5623 individuals with SAKI, 2410 of whom received heparin and 3213 of whom did not. There were significant effects on ICU and 28-day mortality in the overall population with PSM. MSCM further reinforces the efficacy of heparin administration reduces ICU mortality in the general population. Stratification analysis with MSCM showed that heparin administration was associated with decreased ICU mortality at various AKI stages. Heparin use was also associated with reduced 28-day mortality in patients with only female, age >60 years, and AKI stage 3, with HRs of 0.79, 0.77, and 0.60, respectively (*p* < 0.05). E-value analysis suggests robustness to unmeasured confounding.

**Conclusion:** Early heparin therapy for patients with SAKI decreased ICU mortality. Further analysis demonstrated that heparin therapy was associated with reduced 28-day mortality rate in patients only among female, age > 60 years and AKI stage 3.

## Introduction

Sepsis is a life-threatening syndrome characterized by organ dysfunction, including acute kidney injury (AKI), caused by dysregulation of a patient’s response to infection (Koyner, 2021). Studies have shown that the incidence of sepsis associated acute kidney injury (SAKI) ranges from 11% to 64% (Parmar et al., 2009). In a study involving 1177 patients with sepsis in 198 intensive care units in 24 European countries, the incidence of AKI was 51% and the mortality rate was 41% (Vincent et al., 2006). In a retrospective analysis of 146,148 patients in China, the incidence of SAKI was 47.1% (Xu et al., 2015). Other studies have reported a mortality rate of 67%–70.2% in patients with SAKI (Bagshaw et al., 2007; Oppert et al., 2008). SAKI is associated with poor outcomes compared with non-SAKI (Romanovsky et al., 2014), including a significant increase in in-hospital mortality and prolonged intensive care unit (ICU) and hospital length of stay (Bagshaw et al., 2007). The development of AKI predicts a higher mortality rate and consumes a large amount of medical resources, causing great pressure on human and social healthcare.

The inflammatory reaction in the early stage of sepsis can activate the coagulation system, initiate the coagulation cascade reaction, and cause extensive microthrombosis in blood vessels, microvascular disorders, tissue hypoxia, and ischemia, leading to multiple organ dysfunction syndrome (MODS). Heparin is a sulfated polysaccharide polymer that can affect both endogenous and exogenous coagulation pathways. The purpose of anticoagulation therapy in sepsis is to restore the balance between inflammation and coagulation without interfering with the immune defense ability of the body against infection (Semeraro et al., 2015). Many studies have confirmed the therapeutic effects of heparin in sepsis, including the regulation of inflammatory reactions by antagonizing histones (Wildhagen et al., 2014), inhibiting the generation of inflammatory factors (Harada et al., 2006), immune regulation, and vascular protection (Eggimann et al., 2003). Study by Huang and colleagues revealed that heparin administration was also associated with decreased ICU mortality in patients with an SIC score of 4 (HR 0.63, 95% CI 0.45-0.89) (Huang et al., 2023). Whether heparin therapy is associated with reduced mortality in SAKI patients remains controversial. In this retrospective cohort study, we used the Medical Information Mart for Intensive Care IV (MIMIC-IV) database to assess the effectiveness of early heparin in patients with SAKI after ICU admission and to estimate the timing and dosing of heparin.

## Materials and methods

### Data source and study design

We performed a retrospective cohort study using data from the MIMIC-IV (version 1.0), which includes two in-hospital database systems: a custom hospital-wide electronic health record (EHR) and ICU-specific clinical information including de-identified, comprehensive clinical data of patients admitted to the ICUs of Beth Israel Deaconess Medical Center in Boston, Massachusetts, from 2008 to 2019. An individual who has completed the Collaborative Institutional Training Initiative examination (Certification number: 39057014 for author Zhi-peng Zhou) can access the database.

### Participants

There were 382278 patients from the MIMIC-IV database.The inclusion criteria met the definition of Sepsis 3.0 criteria, which was defined as a suspected infection combined with an acute increase in Sequential Organ Failure Assessment (SOFA) score ≥ 2 (Singer et al., 2016) and AKI, which was stipulated in the Kidney Disease: Improving Global Outcomes (KDIGO) guidelines (Ostermann et al., 2020) and MIMIC-IV database to define AKI stages. The exclusion criteria were as follows: age <18 years, ICU stay < 24 h, acquired immune deficiency syndrome, malignant cancer, chronic kidney disease, hepatic failure, use of heparin for dialysis or treatment, use of warfarin and low molecular weight heparin (LMWH), and patients admitted to the ICU more than once. We only included the first ICU admission data from the first hospital stay among patients admitted to hospital multiple times.

### Research procedures and definitions

Data were extracted from MIMIC-IV using Structured Query Language (Jamison, 2003) with Navicat Premium (version 15.0.12) and consisted of age, sex, weight, history of disease (Hypertension, Diabetes, Chronic heart disease, Chronic pulmonary disease), vital signs (heart rate, mean arterial pressure (MAP), respiratory rate, temperature, and oxygen saturation (SPO_2_), laboratory results [white blood cell (WBC) count, platelet count, hemoglobin, International Normalized Ratio (INR), partial thromboplastin time (PTT), and prothrombin time (PT)], acute kidney injury (AKI) stage (within 48h of ICU admission), vasopressor use, mechanical ventilation use, sepsis-induced coagulation (SIC), sequential organ failure assessment (SOFA) score, Simplified Acute Physiology Score II (SAPS II), length of hospital stay, and length of ICU stay.

Laboratory variables of PTT were measured during the ICU stay. The chart times for the measurements and physiological values were extracted from the database. For patients with multiple measurements, the highest daily PTT value was included in the analysis for patients with multiple measurements. None of the screening variables had missing data rates exceeding 5% (Supplementary Table S1). We used the methods of previous studies to analyze this database (sepsis and sepsis-associated acute kidney injury) and analyzed the extracted patient data (Zou et al., 2022).

### Exposure and outcomes

The patients were divided into two groups: the heparin group, comprising patients who received heparin subcutaneously at preventive doses at least once in the ICU, and the control group, comprising patients who received no heparin in the ICU.The primary outcome was ICU mortality. The secondary outcomes included the 7-day, 14-day, 28-day, and in-hospital mortality rates.

### Statistical analysis

The study population was categorized into heparin (intervention) and non-heparin (control) groups according to heparin treatment status during the entire ICU stay, and categorical variables were expressed as percentages. Heparin and non-heparin groups were compared using the Chi-square or Fisher’s exact test, as appropriate. Continuous variables are expressed as mean (standard deviation) or median [interquartile range (IQR)], as appropriate.

Propensity score matching (PSM) was used to account for baseline differences in the probability of receiving heparin (Zhang et al., 2019). PSM measures the probability of a patient being assigned to heparin treatment. In PSM analysis, the heparin group received heparin during the entire ICU stay. Patients in the treatment group were matched to those with untreated patients using nearest-neighbor matching. The standardized mean difference (SMD) was calculated before and after matching to examine whether PSM reduced the differences in pretreatment covariates between the treatment and control groups. Finally, a COX regression model was used to adjust for residual imbalance by including parameters with *p* < 0.05 and potential confounding judged by clinical expertise.

The dose-response relationship between subcutaneous heparin and ICU mortality was also explored by categorizing heparin into subclasses by daily dose (non-heparin, ≤ 5000IU, 5000-7500IU, 7500-10000IU, 10000-12500IU). We also explored the potential for unmeasured confounding between early prophylactic heparin prescriptions and mortality by calculating E-values (Haneuse et al., 2019). The E-value quantifies the required magnitude of an unmeasured confounder that can negate the observed association between heparin therapy and mortality.

Heparin treatment during ICU stay was considered a time-dependent variable in the marginal structural Cox model (MSCM). Potential baseline confounders, such as age, gender, weight, AKI stage, hypertension, diabetes, chronic heart disease, chronic pulmonary disease, vasopressor use, use of mechanical ventilation, SIC, SOFA, and SAPSII, were obtained on day 1 after ICU admission. APTT during the entire ICU stay was included in the model as a time-varying confounding factor, and the parameters of MSCM could be estimated using inverse probability weighting (IPW) to correct for confounding and forms of selection bias such as informative censoring (Robins et al., 2000). By weighting each patient by IPW, two pseudo-populations were created, similar to the baseline and time-dependent confounding factors and different in heparin exposure.

Stratification analysis was conducted to explore whether heparin administration and ICU or 28-day mortality differed across the various subgroups classified by gender, age, SIC, SOFA, vasopressor usage, mechanical ventilation, and AKI stage; two-tailed *p* values < 0.05 were considered statistically significant. All statistical analyses were performed using R 4.2.1 software for Windows.

## Results

### Patient characteristics on the baseline

**FIGURE 1:**
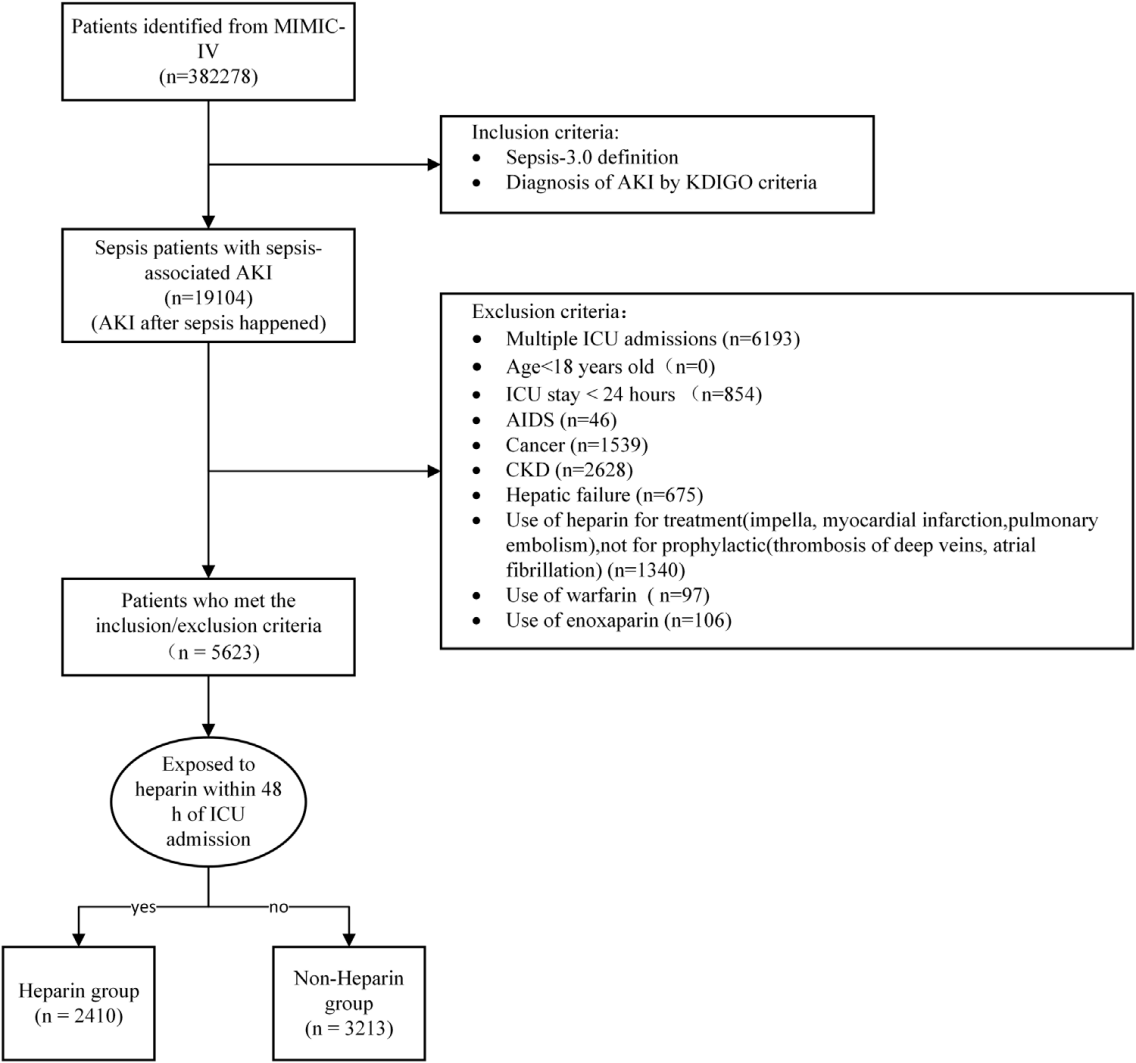
Flow chart of patient selection. Abbreviations:AKI, Acute Kidney Injury; KDIGO, Kidney Disease: Improving Global Outcomes; ICU, Intensive Care Unit; AIDS, Acquired immunodeficiency syndrome; CKD, Chronic Kidney Disease.

The initial search identified 382,278 ICU admissions from the MIMIC-IV database. In total 19,104 patients met the inclusion criteria. After excluding patients who met the exclusion criteria, 5623 eligible patients were enrolled. A total of 2410 patients were administered heparin at least once in the ICU and 3213 patients did not receive heparin treatment (Figure 1). There were no significant differences between the two groups in terms of age, Hypertension, Diabetes, Chronic heart disease, or SOFA score (*p* > 0.05). The proportion of men, percentage of patients with a history of vasopressor use, SIC,weight, SPO_2_, WBC count, PT, APTT, and maximum INR were lower in the heparin group than in the non-heparin group (*p* < 0.05). However, Chronic pulmonary disease, mechanical ventilation use, heart rate, MAP, respiratory rate, temperature, hemoglobin level, minimum platelet count, SAPS II score, hospital stay, and ICU stay were higher in the early heparin group than in the non-heparin group (*p* < 0.05) (Table 1). After PSM, 3,374 patients were enrolled, with 1,687 in each group, except for vasopressor use and ICU stay, and the SMDs of other variables were <0.1, indicating that the baseline variables in the two groups had similar distributions (Table 1; Supplementary Figure S1).

**TABLE 1:** Baseline characteristics of patients with s sepsis-associated acute kidney injury before and after propensity score matching.

		Propensity score matching							
		Before				After			
Characteristics	All cohort	No heparin (n = 3213)	Heparin (*n* = 2410)	*p*-value	SMD	No heparin (*n* = 1687)	Heparin (*n* = 1687)	*p*-value	SMD
Gender, male,n(%)	3171 (56.4)	1973 (61.4)	1198 (49.7)	<0.001	0.237	866 (51.3)	886 (52.5)	0.513	0.024
Age (yr),median(IQR)	67.96 (56.69,79.27)	68.19 (57.89,78.04)	67.47 (54.69,80.69)	0.723	0.040	68.37 (56.25,79.74)	68.18 (54.78,81.06)	0.872	0.009
Weight (kg),median(IQR)	84.00 (70.00,99.00)	85.00 (71.20,98.70)	82.70 (69.00,100.00)	0.042	0.016	84.85 (69.75,100.00)	82.25 (68.50,99.50)	0.226	0.011
Hypertension,n(%)	3412 (60.7)	1934 (60.2)	1478 (61.3)	0.404	0.023	1016 (60.2)	1019 (60.4)	0.944	0.004
Diabetes,n(%)	1580 (28.1)	913 (28.4)	667 (27.7)	0.562	0.016	478 (28.3)	465 (27.5)	0.645	0.017
Chronic heart disease,n(%)	369 (6.6)	221 (6.9)	148 (6.1)	0.294	0.030	107 (6.3)	107 (6.3)	1.000	<0.001
Chronic pulmonary disease,n(%)	1365 (24.3)	703 (21.9)	662 (27.5)	<0.001	0.130	435 (25.8)	428 (25.4)	0.813	0.010
Heart rate (bpm)	85 (77,96)	84 (77,93)	87 (77,99)	<0.001	0.181	86 (77,98)	86 (76,98)	0.766	0.011
MAP (mmHg)	77 (73,83)	77 (73,82)	78 (73,86)	<0.001	0.219	78 (73,85)	78 (72,85)	0.224	0.030
Respiratory rate (bpm)	19 (17,22)	18 (16,21)	19 (17,22)	<0.001	0.265	19 (17,22)	19 (17,22)	0.654	0.026
Temperature (°C)	36.90 (36.62,37.22)	36.88 (36.61,37.13)	36.92 (36.63,37.32)	<0.001	0.153	36.90 (36.65,37,26)	36.90 (36.63,37.30)	0.708	0.007
Spo2 (%)	98 (96,99)	98 (96,99)	97 (96,99)	0.006	0.090	97 (96,99)	97 (96,99)	0.404	0.001
WBC (10³/μl) (IQR)	14.90 (11.20,19.50)	15.20 (11.70,19.40)	14.40 (10.60,19.50)	<0.001	0.079	14.80 (11.10,19.10)	14.50 (10.70,19.60)	0.416	0.010
Hemoglobin (g/L) (IQR)	10.00 (8.60,11.40)	9.70 (8.40,11.00)	10.30 (8.90,12.00)	<0.001	0.312	10.20 (8.70,11.70)	10.20 (8.70,11.70)	0.766	0.018
Minimum platelet (10³/μl) (IQR)	160 (116,218)	147 (109,195)	182 (129,242)	<0.001	0.365	174 (127,227)	171 (119,229)	0.166	0.043
PT(s) (IQR)	14.90 (13.20,17.20)	15.20 (13.60,17.20)	14.30 (12.70,17.20)	<0.001	0.172	14.50 (13.00,17.20)	14.70 (12.90,17.20)	0.403	0.034
APTT(s) (IQR)	32.10 (28.20,37.70)	32.30 (28.50,38.40)	31.80 (27.70,37.50)	0.001	0.041	31.40 (27.60,37.50)	32.30 (27.90,37.50)	0.047	0.009
Maximum INR (IQR)	1.30 (1.20,1.60)	1.40 (1.20,1.60)	1.30 (1.10,1.60)	<0.001	0.179	1.30 (1.20,1.60)	1.30 (1.20,1.60)	0.835	0.029
Vasopressor,n (%)	3304 (58.8)	2069 (64.4)	1235 (51.2)	<0.001	0.269	817 (48.4)	913 (54.1)	0.001	0.114
Ventilation,n (%)	3397 (60.4)	1889 (58.8)	1508 (62.2)	0.004	0.077	991 (58.7)	1028 (60.9)	0.206	0.045
SIC,n (%)	2709 (48.2)	1811 (56.4)	898 (37.3)	<0.001	0.390	688 (40.8)	728 (43.1)	0.174	0.048
AKI stage,n (%)				<0.001	0.215			0.978	0.007
1	1908 (33.9)	1200 (37.3)	708 (29.4)			529 (31.3)	534 (31.6)		
2	2999 (53.3)	1684 (52.4)	1315 (54.6)			914 (54.1)	912 (54.0)		
3	716 (12.8)	329 (10.2)	387 (16.1)			245 (14.5)	242 (14.3)		
SOFA score median (IQR)	5 [4,7]	5 [4,7]	5 [3,8]	0.985	0.013	5 [3,8]	5 [4,8]	0.112	0.021
SAPS II score median (IQR)	37[30,47]	36[29,45]	39[31,49]	<0.001	0.170	39[30,49]	39[30,48]	0.740	0.029
Hospital stays (d) median (IQR)	8.00[5.12,13.79]	6.70[4.84,10.83]	10.27[6.30,17.28]	<0.001	0.354	7.62[5.00,13.91]	9.22[5.82,15.13]	<0.001	0.050
ICU stays (d) median (IQR)	2.88[1.63,5.85]	2.17[1.31,3.76]	4.54[2.49,8.61]	<0.001	0.558	2.84[1.66,5.79]	3.94[2.22,6.89]	<0.001	0.124
Heparin (U) median (IQR)	NA	NA	10000[7500,11562]			NA	10000[7000,11363]		

Abbreviations: IQR, interquartile range; MAP, mean arterial pressure; SPO_2_, oxygen saturation; WBC, white blood cells; PT, prothrombin time; APTT, activated partial thromboplastin time; INR, international normalized ratio; SIC, sepsis-induced coagulopathy; AKI, acute kidney injury; SOFA, sequential organ failure assessment; SAPS II, Simplified Acute Physiology Score II; ICU, intensive care unit; NA, not applicable.

## Outcomes

### Propensity score analysis on primary and secondary outcomes

The prematched crude ICU mortality rate was higher in patients with heparin use than in those without heparin use (11.7% vs. 11.0%, hazard ratio (HR) 0.53, 95% confidence interval (CI) [0.45-0.62] *p* < 0.001). However, after PSM, heparin was associated with reduced ICU mortality (11.7% vs. 14.6%, HR 0.75, 95% CI [0.62-0.92], *p* = 0.005) (Table 2). The 28-day mortality rate in the heparin group was lower than that in the non-heparin group after PSM (postmatched 14.0% vs. 17.4%, HR 0.74, 95% CI [0.59-0.95], *p* = 0.016), and there was no significant difference in 7-day and 14-day and hospital mortality rates between the two groups (*p* > 0.05) (Table 2). Stratification analysis showed an effect only among AKI stage 3 in the primary and secondary outcomes after PSM (Table 3).

**TABLE 2:** Association between heparin use and clinic outcomes in patients with SAKI.

	Propensity score matching cohort (*n* = 3374)				All eligible for propensity score (*n* = 5623)			
Outcomes n (%)	No heparin *n* = 1687	Heparin *n* = 1687	Matched HR (95%)^a^	*p* value	No heparin n = 3213	Heparin n =2410	Adjusted HR (95%CI)^b^	*p* value
Primary								
ICU mortality	247(14.6)	197(11.7)	0.75(0.62,0.92)	0.005	352(11.0)	282(11.7)	0.53(0.45,0.62)	<0.001
Secondary								
7-day mortality	314(12.4)	206(9.2)	0.81(0.58,1.13)	0.209	384(12.0)	120(5.0)	0.95(0.71,1.26)	0.710
14-day mortality	261(15.5)	208(12.3)	0.76(0.58,1.00)	0.051	375(11.7)	287(11.9)	0.95(0.75,1.20)	0.647
Hospital mortality	289(17.1)	241(14.3)	0.85(0.71,1.02)	0.073	405(12.6)	344(14.3)	0.78(0.67,0.90)	0.001
28-day mortality	293(17.4)	237(14.0)	0.74(0.59,0.95)	0.016	413(12.9)	339(14.1)	0.93(0.76,1.14)	0.492

Abbreviations: ARR, absolute risk reduction; HR, hazard ratio; SAKI, sepsis associated acute kidney injury.

^a^ Results of univariable analysis of propensity score matched cohort.

^b^ Adjusted results obtained from the multivariable Cox proportional hazards regression model that included the full cohort.

**TABLE 3:** Primary and secondary outcomes of sepsis-associated acute kidney injury stages.

	HR (95%CI) *p*-value		
Outcomes	Stage 1 (*n* = 1037)	Stage 2 (*n* = 1834)	Stage 3 (*n* = 503)
Primary			
ICU mortality	0.60(0.39,0.93)0.022	0.77(0.57,1.04)0.083	0.59(0.40,0.88)0.009
Secondary			
7-day mortality	0.61(0.37,1.01)0.054	0.90(0.63,1.28)0.543	0.33(0.14,0.78)0.011
14-day mortality	0.68(0.43,1.06)0.086	0.87(0.63,1.19)0.371	0.37(0.18,0.73)0.005
Hospital mortality	0.83(0.58,1.21)0.346	0.86(0.65,1.12)0.259	0.68(0.50,0.94)0.018
28-day mortality	0.78(0.52,1.18)0.236	0.81(0.60,1.10)0.174	0.40(0.21,0.74)0.004

Abbreviations: ICU, intensive care unit;HR, hazard ratio; CI, confidence interval.

### Marginal structural cox model and stratification analysis for ICU mortality

**FIGURE 2:**
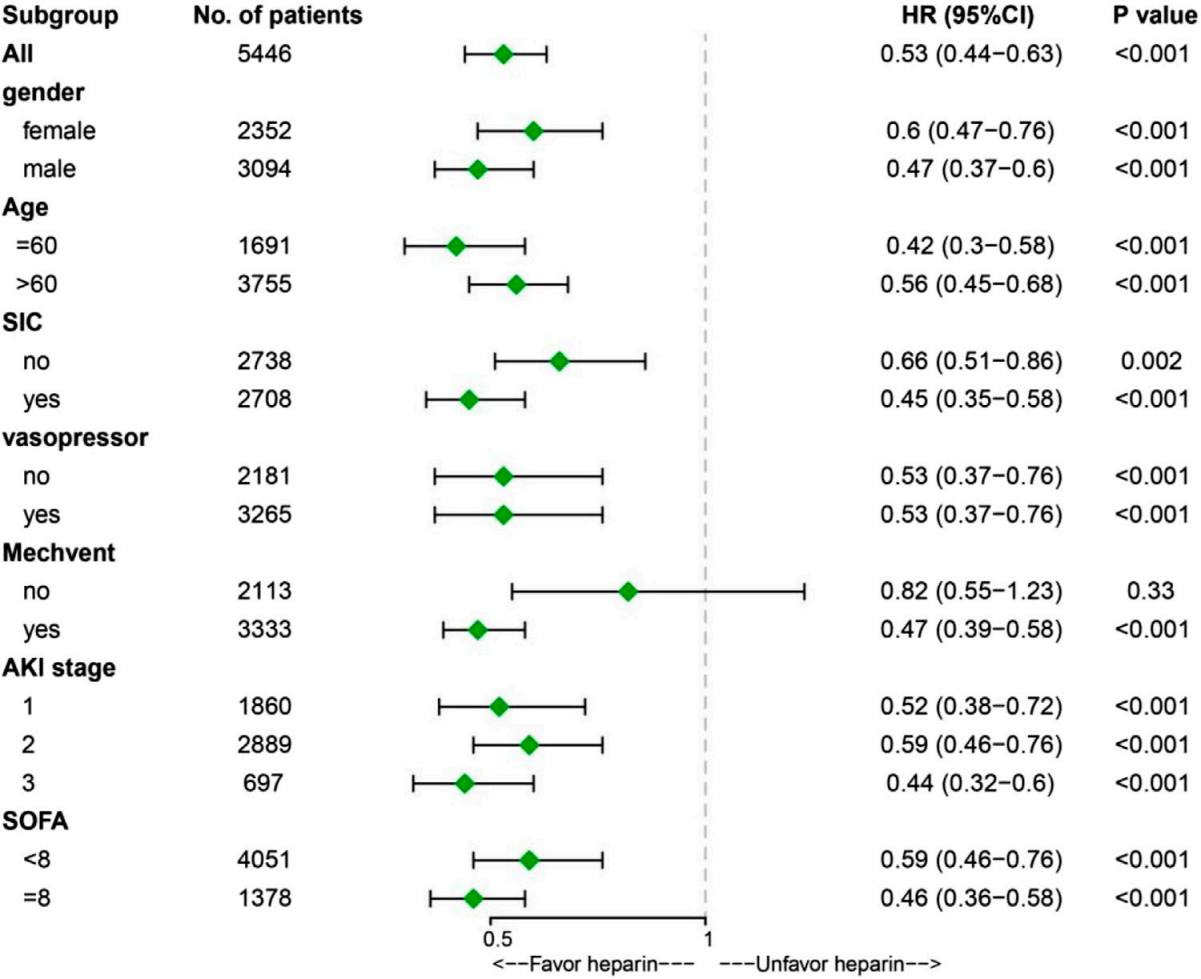
Results of ICU mortality in overall population with marginal structural Cox model and stratification analysis. Abbreviations: SIC, sepsis-induced coagulopathy; Mechvent, mechanical ventilation; AKI, acute kidney injury; SOFA, Sequential Organ Failure Assessment; HR, Hazard Ratio; CI, confidence internal; ICU, Intensive Care Unit.

Time-varying confounding and heparin treatments were included in the MSCM. The MSCM results showed that heparin administration was associated with significantly improved ICU mortality (HR 0.53, 95% CI 0.44-0.63, *p* < 0.001) in the overall population (Figure 2). Stratification analysis with MSCM further showed that heparin administration was associated with decreased ICU mortality at different AKI stages, regardless of gender, age, mechanical ventilation, sequential organ failure assessment (SOFA) score, and history of SIC and vasopressor use (Figure 2).

### Logistic regression model and stratification analysis of the 28-day mortality

**FIGURE 3:**
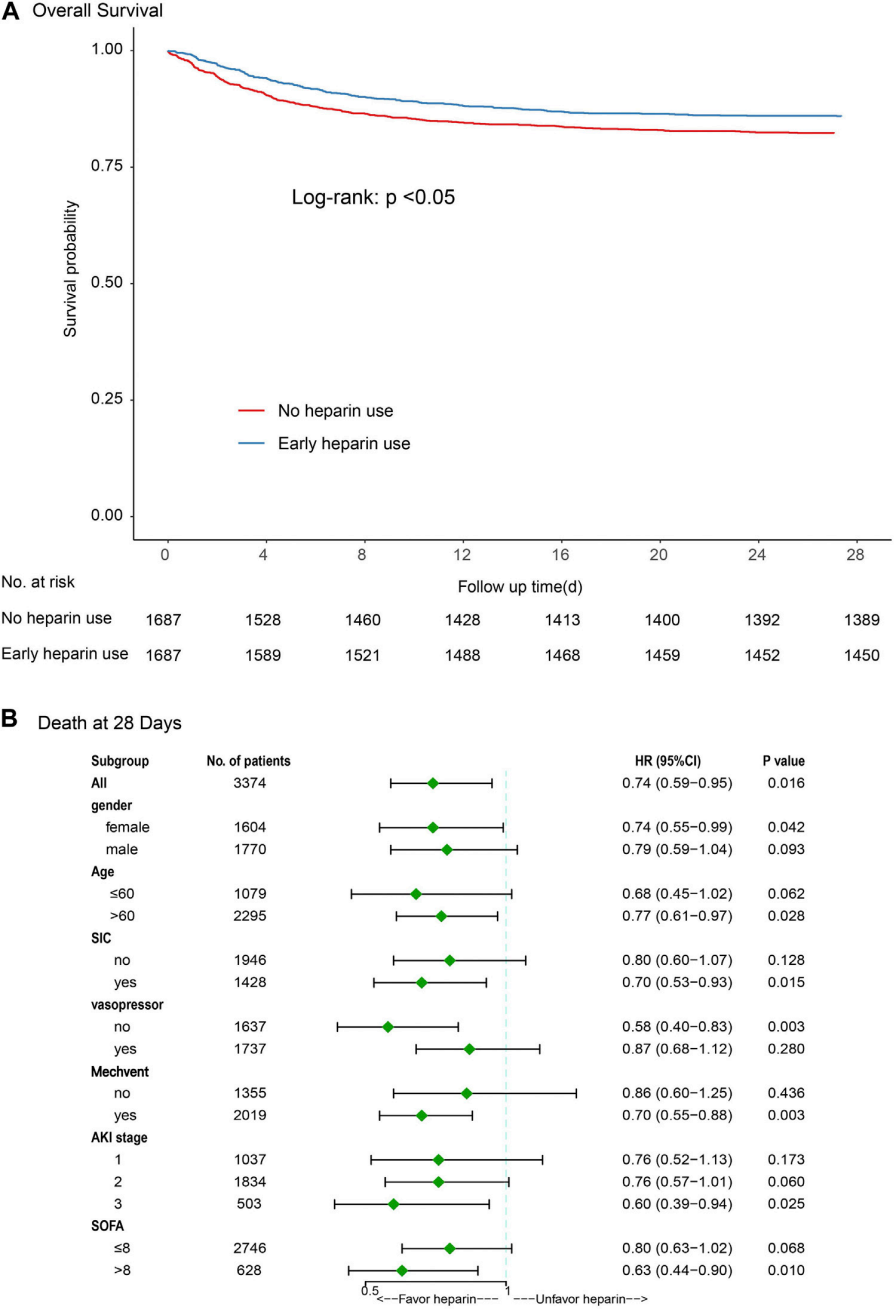
Results of 28-day mortality in overall population with logistic regression model and stratification analysis [**(A)** Kaplan-Meier curves; **(B)** Subgroup analysis]. Abbreviations: SIC, sepsis-induced coagulopathy; MV, mechanical ventilation; AKI, acute kidney injury; SOFA, Sequential Organ Failure Assessment.

Kaplan-Meier curves showed a significant difference between heparin use and non-heparin use after PSM (*p* < 0.05) (Figure 3A). Subgroup analysis showed that heparin use was significantly associated with reduced 28-day mortality in patients with only female, age >60 years, sepsis-induced coagulopathy (SIC), non-vasopressor use, mechanical ventilation, AKI stage 3, and SOFA score ≥ 8, with HRs of 0.79, 0.77, 0.70, 0.58, 0.70, 0.60, and 0.63, respectively (*p* < 0.05) (Figure 3B).

### Curve fitting and subgroup analysis

There was a nonlinear relationship between heparin therapy and ICU mortality with curve fitting (Supplementary Figure S2). The outcomes also showed that receiving 10000–12500 IU a day in patients with AKI stage 1, 2, and 3 was associated with decreased risk of ICU mortality as compared with the non-heparin group, similar outcomes were showed for receiving 12500–15000 IU a day in patients with AKI stage 1 and 3. For receiving less than 5000 IU, 5000–7500 IU and 7500–10000 IU a day, there was no significant difference in ICU mortality as compared with the non-heparin group (*p* > 0.05) (Table 4).

**TABLE 4:** Dose-response relationship between heparin and ICU mortality in SAKI patients.

Daily heparin usage (non-heparin group as reference)	HR (95%CI) *p*-value			
	Full cohort (*n* = 3374)	Stage 1 (*n* = 1037)	Stage 2 (*n* = 1834)	Stage 3 (*n* = 503)
0U < *×* ≤ 5000U	1.25 (0.89, 1.77) 0.202	1.57 (0.74, 3.34) 0.240	1.19 (0.69, 2.05) 0.543	1.33 (0.76, 2.33) 0.313
5000U < *×* ≤ 7500U	0.69 (0.46, 1.02) 0.061	0.63 (0.29, 1.37) 0.240	0.72 (0.40, 1.28) 0.264	0.87 (0.41, 1.83) 0.715
7500U < *×* ≤ 10000U	0.77 (0.57, 1.02) 0.069	0.93 (0.54, 1.59) 0.783	0.81 (0.54, 1.23) 0.324	0.59 (0.33, 1.08) 0.088
10000U < *×* ≤ 12500U	0.39 (0.27, 0.56) <0.001	0.40 (0.19, 0.82) 0.013	0.52 (0.31, 0.85) 0.009	0.26 (0.13, 0.54) <0.001
12500U < *×* ≤ 15000U	0.50 (0.31, 0.81) 0.005	0.30 (0.11, 0.87) 0.026	0.93 (0.51, 1.66) 0.795	0.30 (0.09, 1.00) 0.050
*p* value for trend	<0.001	0.002	0.021	<0.001

Abbreviations: ICU, intensive care unit; HR, hazard ratio; CI, confidence interval; SAKI, sepsis-induced acute kidney injury.

### Sensitivity analysis

Significant known and measured risk factors for ICU mortality after PSM within the multivariable Cox-proportional hazard model included age (HR, 1.01 [95%CI,1.01-1.02]), heart rate(HR, 1.01 [95%CI,1.00-1.01]), respiratory rate (HR, 1.10 [95%CI,1.08-1.12]), WBC(HR, 1.02 [95%CI,1.01-1.03]), PT(HR, 1.02 [95%CI,1.01-1.02]), APTT(HR, 1.01 [95%CI,1.00-1.02]), INR(HR, 1.14 [95%CI,1.10- 1.19]), vasopressor use (HR, 1.94 [95%CI,1.55-2.42]), mechanical ventilation use(HR, 1.19 [95%CI,0.94-1.50]), SIC (HR, 1.67 [95%CI, 1.39-2.01]), SAPSII (HR, 1.04 [95%CI,1.04-1.05]), SOFA (HR, 1.14 [95%CI,1.11-1.16]) (Supplementary Table S2).

We generated an E-value to assess the sensitivity to unmeasured confounding factors (https://www.evalue-calculator.com/evalue/). The primary findings were robust, unless an unmeasured confounder existed with a lower relative risk of ICU mortality, with an HR > 2.00 (upper limit 3.00), meaning that residual confounding could explain the observed association if there exists an unmeasured covariate having a relative risk association >2.00 with both ICU mortality and prophylactic heparin prescriptions. Therefore, it is unlikely that an unmeasured or unknown confounder would have a substantially greater effect on ICU mortality (relative risk > 2.00) than known risk factors.

## Discussion

Our results showed that early heparin therapy improved the survival outcomes of patients with SAKI. Stratification analysis with MSCM showed that heparin administration was associated with decreased ICU mortality in different AKI stages, regardless of gender, age, mechanical ventilation, SOFA score, and the use of SIC and vasopressors. Heparin use was also significantly associated with reduced 28-day mortality in the logistic regression model in female patients, age >60 years, SIC, non-vasopressor use, mechanical ventilation, AKI stage 3, and SOFA score ≥8.

In our study, the early use of heparin was also significantly associated with reduced in-hospital mortality in patients with SAKI, consistent with previous studies of heparin use in sepsis (Zou et al., 2022). The prematched crude ICU mortality rate was higher in patients with heparin use than in those without heparin use (11.7% vs. 11.0%, HR 0.53, 95% CI 0.45-0.62, *p* < 0.001) and after PSM, heparin was associated with reduced ICU mortality (11.7% vs. 14.6%, HR 0.75, 95% CI [0.62-0.92], *p* = 0.005) The underline reason for this is ICU mortality was represented by percentage, while HR increased the effect of survival time on the outcome. We can see that heparin extended the length of ICU stay of patients (2.84 VS 3.94 *p* < 0.001) from in Table 1. The pathophysiology of acute kidney injury in sepsis is complex, including organ ischemia and systemic hemodynamic changes, as well as kidney inflammation and response to various septic mediators, including inflammation, microcirculatory dysfunction, ischemia-perfusion injury, and cellular adaptation to injury (De Backer et al., 2011; Gomez et al., 2014; Honore et al., 2015; Peerapornratana et al., 2019). Severe changes in systemic microvascular distribution caused by sepsis include a significant decrease in capillary density, a decrease in the proportion of capillaries with continuous flow, and an increase in the proportion of capillaries with intermittent or no flow. Based on Inflammatory and Coagulation Indicators (platelet, serum procalcitonin, prothrombin time activity) may be a robust predictor for the SAKI in patients, which providing information for timely and efficient intervention (Xin et al., 2022).

Heparin is the oldest and most widely used anticoagulant worldwide. It has been used in clinical practice for 80 years since 1935 (Wang et al., 2022). Heparin, except for its anticoagulant properties, also has anti-inflammatory activity and resistance to complement and regulate the action, such as all kinds of proteases, and its mechanism has two types: one type is adjusted by combining plasma soluble ligand, and the other is through a combination of cell surface receptors or adjusted macromolecules, which have potential effects on downstream signaling pathways. Heparin can inhibit the activation of inflammatory cells and responses by binding to inflammatory mediators and enzymes (Beurskens et al., 2020). Research in 2004 showed that NETosis is a process of density-chromatin formation consisting of nuclear DNA-histone scaffold, called NETs, that responds to a trigger (usually a pathogen), NETs are a major component of arterial and venous thrombosis, as demonstrated in several *in vivo* models and patients, heparin protects against NETosis (von Brühl et al., 2012). Histones are cytotoxic in extracellular presence and are closely related to endothelial dysfunction; sepsis, kidney ischemia, necrosis of tubular epithelial cells release histones into the extracellular space, and renal vascular endothelial and renal tubular epithelial cells produce a dose-dependent toxicity, increased vascular permeability, and neutrophils to renal parenchyma across endothelial migration. Heparin caused by severe inflammation cell damage during extracellular histones have strong affinity, which can reduce this phenomenon (Allam et al., 2012; Saffarzadeh et al., 2012). In addition, neutrophils are known to be responsible for the development of AKI, and neutrophil-derived Heparin-binding Protein (HBP) also plays an important role in sepsis-induced AKI (Fisher et al., 2017). HBP has been shown to increase endothelial permeability, cause renal bleeding and vascular leakage, and induce inflammation in renal tubular cells (Gomez et al., 2014). Studies have shown that heparin may block GAG-binding sites on HBP and prevent their association with cell-surface GAGs, thereby attenuating HBP-induced renal vascular leakage and inflammation (Fisher et al., 2017). But there is no recommendation in the international sepsis guidelines on whether they require anticoagulation treatment in patients without venous thromboembolism (VTE) (Evans et al., 2021). The possible reason may be associated with the heterogeneity of sepsis, and it is necessary to pay attention to the onset stage of sepsis and heparin dosage for clinicians.

The optimal dosage of heparin in patients with sepsis remains controversial. In our study, 1000–20000 IU/day was shown to reduce adverse outcomes and improved patient prognosis. Heparin-related side effects, including bleeding and thrombocytopenia, should not be ignored. In some related studies, the use of intravenous heparin was not associated with increased gastrointestinal or intracranial bleeding (Zarychanski et al., 2008; Liu et al., 2014). However, it is necessary to closely observe and monitor relevant indicators when using them.

In our stratified analysis, heparin use in women was significantly associated with reduced 28-day mortality, which may be mediated by differences in steroid hormone levels (O'Brien et al., 2019). Men may be more susceptible to infection than women, not only because androgens reduce immunity but also because steroid hormones affect disease-fighting genes and behavior (Klein, 2000). Elderly patients are independent risk factors for venous thromboembolism, and it has been reported that pharmacodynamic changes in sensitivity to drugs are better in the elderly (Mangoni and Jackson, 2004); for example, besides antithrombin unfractionated heparin combined with many plasma proteins, these factors may help heparin in the elderly with unpredictable pharmacokinetic and pharmacodynamic properties (Dorobantu and Bogdan, 2016). Heparin is a glycosaminoglycan with anticoagulant and anti-inflammatory effects (Robertson, 2006). Studies have shown that in patients with SAKI with coagulopathy, the use of heparin can improve the prognosis of patients, which is related to the anticoagulant and non-anticoagulant effects of heparin (Li et al., 2011). Heparin is used as an anticoagulant, and its main effect is to increase the inactivation of factor Xa and thrombin mediated by antithrombin, thus effectively limiting the production of thrombin (Evans et al., 2021). As thrombin production is closely related to inflammation, heparin also plays an anti-inflammatory role. Heparin neutralizes endotoxins and increases serum tumor necrosis factor-binding protein-1, directly limiting coagulation and inflammation activation (Schultz and Becker, 1967).

Notably, our results must be interpreted in the context of the limitations of our study. First, it was a retrospective study. Due to the large time span, there may be measurement bias; therefore, we used PSM analysis to reduce this bias. Second, some patient variables were not extracted from the database, which may have led to confusion. Third, due to the large time span of the data, sepsis-related definitions have changed in clinical practice studies, which may lead to the results not being generalized to current practice. Lastly, according to the KDIGO criteria, AKI can be classified into two categories: persistent AKI, defined as continuing AKI for more than 48 h from onset, and transient AKI, where there is complete reversal of AKI within 48 h of onset (Cardoso et al., 2022). Thus, additional research is needed to assess the effectiveness of heparin in both transient and persistent AKI.

## Conclusion

The present study suggests that early heparin administration to patients with SAKI who received 10000–15000 IU/day appears to be associated with improved ICU mortality at different AKI stages, regardless of gender, age, mechanical ventilation, sequential organ failure assessment (SOFA) score, and history of SIC and vasopressor use. Patients with female, age >60 years, sepsis-induced coagulopathy (SIC), non-vasopressor use, mechanical ventilation, AKI stage 3, and SOFA score ≥ 8 who received heparin had decreased 28-day mortality. A prospective randomized controlled study should be conducted to further verify these findings.

## Data Availability

The datasets presented in this study can be found in online repositories. The names of the repository/repositories and accession number(s) can be found below: these data are available at https://mimic-iv.mit.edu/.
