# Supplementary material for: Impact of early heparin therapy on mortality in critically ill patients with sepsis associated acute kidney injury: a retrospective study from the MIMIC-IV database
Source: Front Pharmacol. 2024 Jan 11;14:1261305. doi: 10.3389/fphar.2023.1261305 (PMC10808568; doi:10.3389/fphar.2023.1261305)
Supplement: Supplementary file 1 [file Table1.DOCX]

***Supplementary materials***

Table S1. Missing number (%) for included variables in the datasets

| **Variables** | Missing (%) |
| --- | --- |
| Age | 0 (0) |
| Gender | 0 (0) |
| weight | 83（1.4%） |
| hypertension | 0 (0) |
| diabetes | 0 (0) |
| CHD | 0 (0) |
| CPD | 0 (0) |
| heart_rate | 3 (0.05%) |
| MAP | 21 (0.37%) |
| RR | 3 (0.05%) |
| temperature | 357 (6.3%) |
| SpO2 | 4(0.07%) |
| WBC | 13(0.23%) |
| PT | 249 (4.4%) |
| APTT | 276(4.9%) |
| INR | 249 (4.4%) |
| AKI_stage | 0 (0) |
| vasopressor | 0 (0) |
| MV | 0 (0) |
| SIC | 18 (0.3%) |
| SAPS II | 0 (0) |
| SOFA | 18 (0.3%) |
| 7-days mortality | 0 (0) |
| 14-days mortality | 0 (0) |
| 28-days mortality | 0 (0) |
| ICU mortality | 0 (0) |
| Hospital mortality | 0 (0) |
| Length of ICU stay | 0 (0) |
| Length of hospital stay | 0 (0) |

Abbreviations: CHD,Chronic heart disease; CPD,Chronic lung disease; MAP, mean arterial pressure; RR,Respiratory rate; WBC, white blood cell; PT, prothrombin time; APTT, activated partial thromboplastin time; INR, international normalized ratio;AKI, acute kidney injury; SIC, sepsis-induced coagulopathy; SOFA, sequential organ failure assessment; SAPS II, simplified acute physiology score II

Table S2. Multivariable cox regression model after propensity score matching in critically ill patients with SAKI

| Variables | Univariate Cox HR(95%CI) | P-value | Multivariable cox HR(95%CI) | P-value |
| --- | --- | --- | --- | --- |
| Male | 1.09 (0.76,1.57) | 0.642 | 1.01 (0.84, 1.21) | 0.921 |
| Age | 1.00 (0.99, 1.02) | 0.609 | 1.01 (1.01, 1.02) | <0.001 |
| Weight | 1.00 (0.99,1.01) | 0.637 | 0.99 (0.99, 0.99) | <0.001 |
| Hypertension | 0.83 (0.57, 1.21) | 0.322 | 0.93 (0.77, 1.12) | 0.439 |
| Diabete | 1.20 (0.78, 1.85) | 0.412 | 0.88 (0.71, 1.09) | 0.233 |
| CHD | 0.47 (0.23, 0.99) | 0.046 | 1.20 (0.81, 1.77) | 0.363 |
| CPD | 0.98 (0.64,1.53) | 0.944 | 0.86 (0.69, 1.07) | 0.177 |
| Heart rate | 1.00 (0.99,1.02) | 0.389 | 1.01 (1.00, 1.01) | 0.002 |
| MAP | 0.99 (0.97,1.01) | 0.463 | 0.99 (0.98, 1.00) | 0.013 |
| Respiratory rate | 1.13 (1.09,1.18) | <0.001 | 1.10 (1.08, 1.12) | <0.001 |
| Temperature | 0.68 (0.56,0.83) | <0.001 | 0.64 (0.57, 0.70) | <0.001 |
| SPO2 | 0.98 (0.93,1.02) | 0.305 | 0.90 (0.87, 0.93) | <0.001 |
| WBC | 0.99 (0.97, 1.01) | 0.404 | 1.02 (1.01, 1.03) | <0.001 |
| PT | 1.04 (0.95,1.13) | 0.448 | 1.02 (1.01, 1.02) | <0.001 |
| APTT | 1.01 (1.00, 1.02) | 0.010 | 1.01 (1.01, 1.01) | <0.001 |
| INR | 0.86 (0.34,2.18) | 0.754 | 1.14 (1.10, 1.19) | <0.001 |
| Vasopressor | 1.27 (0.67, 2.38) | 0.464 | 1.94 (1.55, 2.42) | <0.001 |
| MV | 0.81 (0.47, 1.38) | 0.436 | 1.19 (0.94, 1.50) | 0.142 |
| SIC | 0.87 (0.55,1.40) | 0.576 | 1.67 (1.39, 2.01) | <0.001 |
| SAPSII | 1.03 (1.01,1.05) | 0.001 | 1.04 (1.04, 1.05) | <0.001 |
| SOFA | 0.97 (0.90, 1.05) | 0.480 | 1.14 (1.11, 1.16) | <0.001 |

Abbreviations: HR, hazard ratio; CHD,Chronic Heart Disease; CPD,Chronic pulmonary disease; MAP, mean arterial pressure; SPO2, saturation pulse oximetry; WBC,white blood cell, PT,prothrombin time, APTT,activated partial thromboplastin time, INR,international normalized ratio, MV, mechanical ventilation; SIC,sepsis-induced coagulopathy, SAPS II, simplified acute physiology score II, SOFA, sequential organ failure assessment. A Cox proportional hazards model showed that age, HR, RR, WBC, PT, APTT, INR, AKI stage 3, vasopressor use, SIC, SAPS II, and SOFA score were independent risk factors for ICU mortality after propensity score matching.


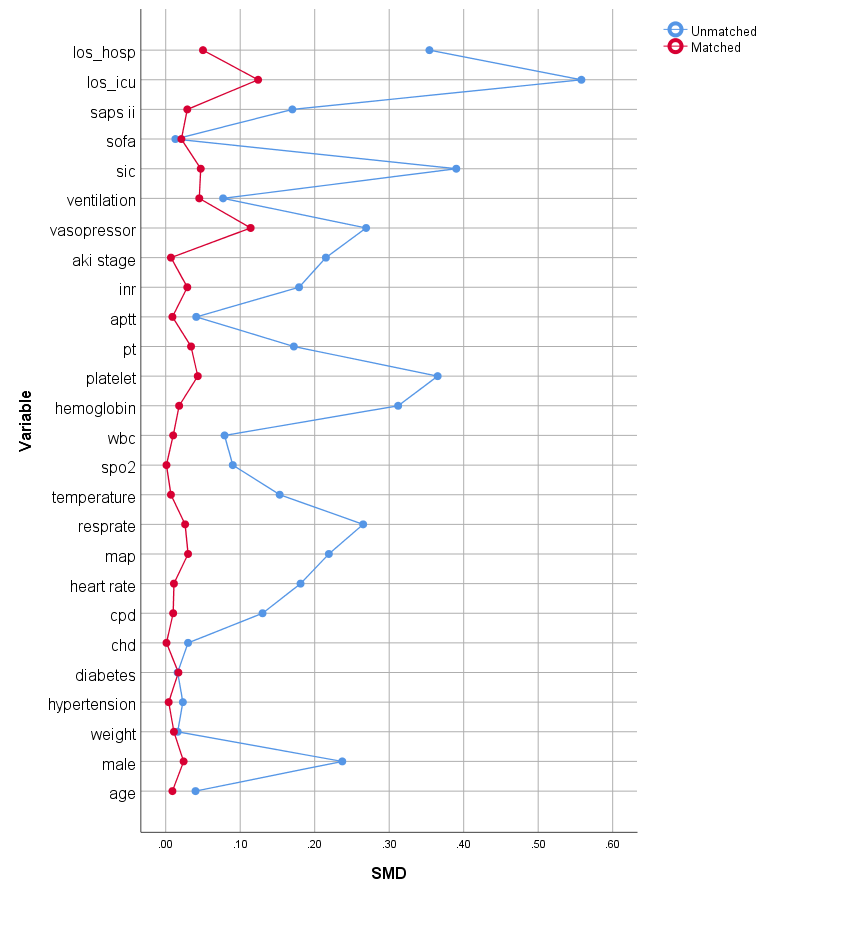


Fig. S1. Standardized mean difference (SMD) of variables before and after propensity score matching.


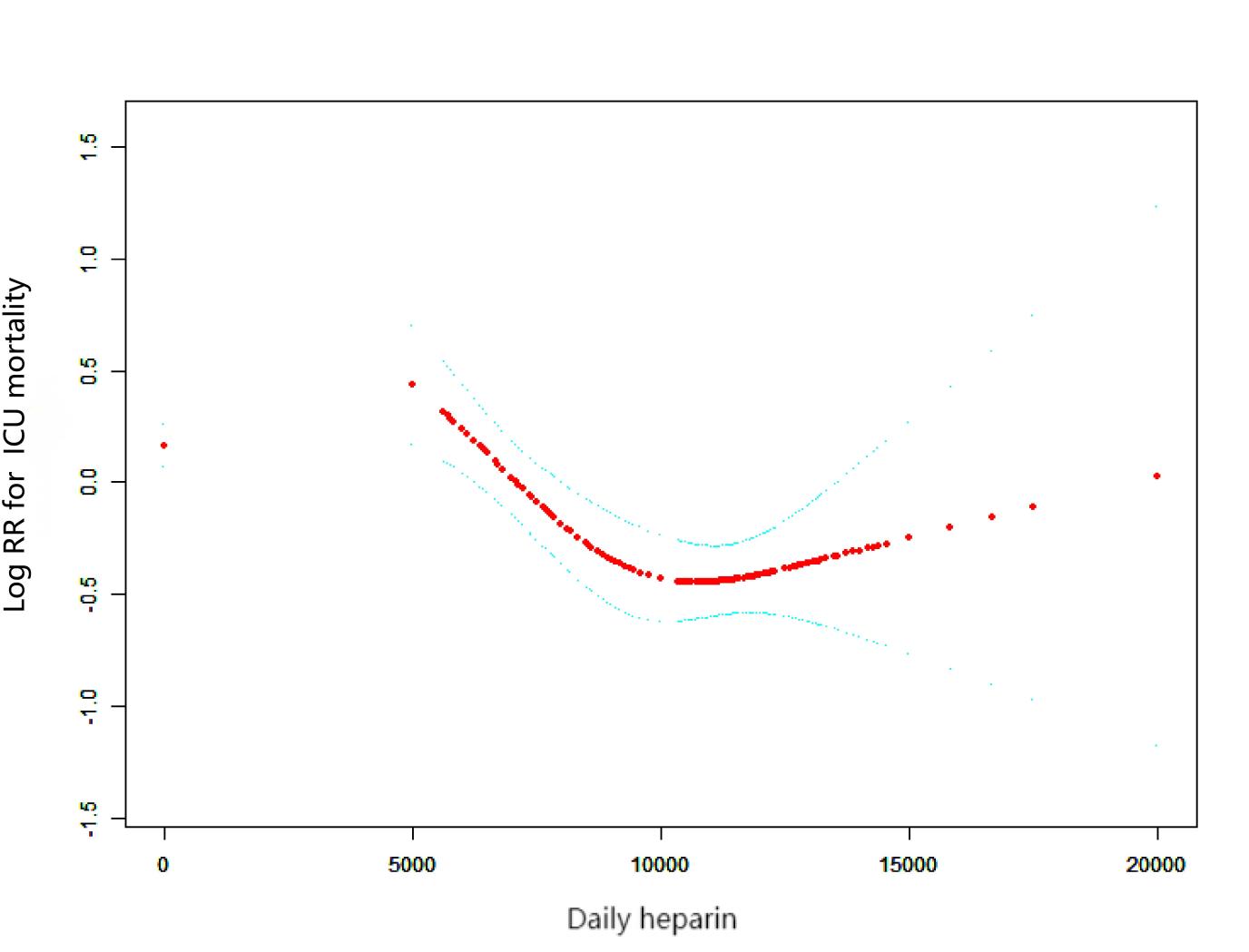


Fig. S2. Association of prophylactic heparin administration with RR for ICU mortality
